# A commentary on ‘Can cardiovascular reserve index (CVRI) on arrival to the trauma unit detects massive hemorrhage and predicts developing hemorrhage? observational prospective cohort study’

**DOI:** 10.1097/JS9.0000000000001275

**Published:** 2024-03-04

**Authors:** Tingfen Han, Wanyi Zhang, Sheng Zhang

**Affiliations:** a Department of Traditional Chinese Medicine, Taizhou Hospital of Zhejiang Province Affiliated to Wenzhou Medical University; b Department of Intensive Care Unit, Taizhou Hospital of Zhejiang Province Affiliated to Wenzhou Medical University, Taizhou; c Department of Emergency Medicine, The Third Affiliated Hospital of Zhejiang Chinese Medical University, Hangzhou, People’s Republic of China

*Dear Editor*,

Hemorrhage is one of the leading causes of death after trauma, and compensatory mechanisms of the body may delay the early diagnosis^1^. Shaya *et al*.^2^ conducted a prospective cohort study to assess the immediate detection and future prediction capabilities of can cardiovascular reserve index (CVRI) in trauma casualties upon arrival. Analyzing 71 adult injured individuals, the study concluded that CVRI could be a useful additional tool for evaluating trauma casualties, complementing conventional measurement variables such as lactate, hemoglobin, vital signs, and shock index.

Commendably, this study focused on evaluating the feasibility of CVRI in multicasualty and prehospital settings, addressing a less explored area in traumatic hemorrhage. Rigorous study methods, including clear inclusion and exclusion criteria, offer valuable insights into the study’s completeness. We highly appreciate while we acknowledge the authors’ research efforts, several details require further discussion.

Firstly, expanding the sample size is advisable. An ideal predictive indicator is expected to be applied to various populations, which requires incorporating different populations for validation. For example, Nelson^3^ conducted a retrospective cohort analysis of 1084 severely injured patients and found that obese trauma patients have an increased risk of early hypovolemic shock. As this is an observational study, the defined outcomes and covariates are limited to 71 patients (after exclusion). For greater universality, we suggest expanding the sample size to increase patient diversity and promoting subgroup analyses based on demographics, baseline characteristics, or comorbidities.

Secondly, providing detailed information on the hemoglobin (Hb) measurement method and premeasurement medical treatments is crucial. Hb is indispensable for determining the current status and transfusion needs of patients with hemorrhage. The criterion for developing hemorrhage (DH) involves a reduction of Hb >1 g/dl within 12 h, requiring accurate Hb values. Hb can be divided into peripheral venous blood and arterial blood, and different sources lead to different results^4^. In addition, although the authors mentioned continuous Hb measurement, factors like fluid resuscitation, vasopressors, and blood transfusions (<6 O+) before a certain measurement could influence. The authors should explicitly mention the measurement interval and clarify whether measurements were conducted on time or on demand to improve result accuracy.

Thirdly, describing the respiratory support of enrolled patients is essential. Respiratory rate is one of the three parameters of CVRI. Theoretically, the effects of mechanical and nonmechanical ventilation on respiratory rate differ, so the results of calculating CVRI will also be different. In previous studies on animal exsanguination models, Uri Gabbay *et al*.^5^ clearly stated that all 17 swine were artificially ventilated. Severe and multiple injuries require tracheal intubation. This study excluded casualties undergoing cardiopulmonary resuscitation upon arrival, but it remains unclear if enrolled patients underwent mechanical ventilation, requiring further clarification.

This exploratory study examining the predictive ability of CVRI upon arrival to the trauma unit for traumatic hemorrhage holds significant clinical implications. Addressing the aforementioned recommendations would substantially enhance its clinical applicability and relevance.

## Ethical approval

This manuscript is a comment. Do not need ethical approval.

## Consent

This manuscript is a comment. Do not need consent.

## Sources of funding

Medical and Health Science and Technology Project, Zhejiang Province (2022KY436). Taizhou Technology Project, Zhejiang Province (21ywa03).

## Author contribution

T.H.: investigation and writing – original draft; W.Z.: conceptualization; S.Z.: supervision, funding acquisition, and writing – review and editing.

## Conflicts of interest disclosure

The authors declare that they have no conflicts of interest.

## Research registration unique identifying number (UIN)

None.

## Guarantor

Sheng Zhang.

## Data availability statement

Not applicable.
